# NSPCC

**Published:** 1965-04

**Authors:** Arthur Morton

**Affiliations:** *Director*. 1 Riding House Street, London, W.1


### *Letter to the Editor*

Dear Sir,

**NSPCC—AN URGENT APPEAL**

The NSPCC is in need of special and immediate help and I would like, through your columns, to appeal to your readers for support in caring for needy British children. We have recently found it necessary to extend the Training period for our Inspectors from six months to one year. There was also an urgent need to establish an Emergency Relief & Welfare Department, as well as a Research Department in Child Welfare. These facts, coupled with the expiry of the lease on our old and inadequate Headquarters building, forced us into the additional heavy expense of a move to new offices. Our new Headquarters has a 940 year lease and accommodation for our foreseeable needs, but we must now raise £250,000—in addition to our normal income.

The NSPCC is an entirely voluntary organization. Last year, the Society helped over 120,000 children of whom 75,000 had been neglected and over 9,000 had been the victims of assault or illtreatment. It is a tragic fact that almost half the number of children helped by the Society are under 5 years of age.

These figures speak for themselves and I hope they will move your readers to give special help to the NSPCC in its present need. Donations would be greatly appreciated as would the proceeds of special efforts run by Local Organizations.

Volunteers are needed in many districts to act as Stewards for our Brick Scheme. Stewards are asked to place ten personal collecting boxes amongst their friends and neighbours, to collect the contributions periodically and to pass the money to the Local Committee of the NSPCC through which they will be working. We hope that holders of Brick Boxes will try to contribute £1 to the NSPCC.

Many appeals are addressed to the generous British public but I earnestly hope that this one may find a special place in their hearts because the need is great—and urgent! Any contributions which your readers may send to me will be promptly and gratefully acknowledged as will all offers of help and, when it is appropriate to do so, readers and Local Organizations who kindly offer us their help will be put in touch with our Local Committee.

Yours sincerely,

(Rev.) Arthur Morton,
*Director.*

1 Riding House Street, London, W.1.

